# Challenges and Emerging Technologies within the Field of Pediatric Actigraphy

**DOI:** 10.3389/fpsyt.2014.00099

**Published:** 2014-08-21

**Authors:** Barbara Galland, Kim Meredith-Jones, Philip Terrill, Rachael Taylor

**Affiliations:** ^1^Department of Women’s and Children’s Health, University of Otago, Dunedin, New Zealand; ^2^Department of Medicine, University of Otago, Dunedin, New Zealand; ^3^School of Information Technology and Electrical Engineering, The University of Queensland, Brisbane, QLD, Australia

**Keywords:** accelerometer, actigraphy, child, infant, pediatric, physical activity, sleep, wake

## Abstract

Actigraphy as an objective measure of sleep and wakefulness in infants and children has gained popularity over the last 20 years. However, the field lacks published guidelines for sleep–wake identification within pediatric age groups. The scoring rules vary greatly and although sensitivity (sleep agreement with polysomnography) is usually high, a significant limitation remains in relation to specificity (wake agreement). Furthermore, accurate algorithm output and sleep–wake summaries usually require prior entry from daily logs of sleep–wake periods and artifact-related information (e.g., non-wear time), involving significant parent co-operation. Scoring criteria for daytime naps remains an unexplored area. Many of the problems facing accuracy of measurement are inherent within the field of actigraphy itself, particularly where sleep periods containing significant movements are erroneously classified as wake, and within quiet wakefulness when no movements are detected, erroneously classified as sleep. We discuss the challenges of actigraphy for pediatric sleep, briefly describe the technical basis and consider a number of technological approaches that may facilitate improved classification of errors in sleep–wake discrimination.

## Introduction

No single index or assessment can capture the complex nature of infant and child sleep patterns and behaviors accurately. Questionnaires, daily sleep logs, full or abbreviated polysomnography (PSG), videosomnography, and actigraphy represent the range of tools to capture this, each with its own limitations. Despite measurement techniques for sleep having undergone a boom in the industry with the advent of actigraphy in the early 90s, the field has virtually stalled with few significant advances in performance of actigraphy over the last decade. Actigraphy hardware is constantly evolving (1), but limitations in software persist in regard to the reliability of identifying quiescent periods of wake from sleep. A wakefulness detection specificity of <60% was detected in more than half of pediatric validation studies reviewed by Meltzer and colleagues (2). Such errors affect all derived sleep–wake parameters.

Actigraphy devices are useful for assessing habitual sleep–wake cycles, sleep quality and maintenance in healthy children, and within the clinical field, useful for discriminating between circadian disorders (3), identifying insomnia and hypersomnia, documenting treatment response in children with sleep disorders and with behavioral disorders, and identifying significant sleep disturbance in children with chronic medical conditions (4).

This review does not aim to rewrite the current status of actigraphy in pediatric sleep, recently and comprehensively covered by others (2, 5, 6). Rather, we present an overview of the challenges facing actigraphy within the pediatric field, we then describe the technical basis and common algorithms used to score sleep before reviewing a number of technological approaches, which may facilitate improved classification of errors in sleep–wake detection. Lastly, we consider an approach to estimate both sleep and physical activity intensity within the one device.

## Challenges for Actigraphy within Pediatric Sleep

The normal pattern of infant and toddler sleep with several wakings occurring overnight can be viewed as similar to that of the adult sleep pattern where disturbed sleep is a feature. Problems recognized within the earliest records of actigraphy in studying patients with disturbed sleep (7) still hold today. For this reason, actigraphy does not have strong standing as a clinical evaluation tool within common clinical conditions where sleep is fragmented. For example, actigraphy underestimates total sleep time in sleep disordered breathing (8) and grossly overestimates limb movement events in periodic limb movement disorder in children (9).

Despite the widespread use of actigraphy for sleep assessment, there is no standard in actigraphy sleep scoring rules comparable to the Rechtschaffen and Kales (10) sleep scoring rules for PSG. Standardization of scoring is made difficult by the variety of proprietary hardware available (each utilizing different sampling rates and on-board processing), and that this hardware is typically coupled with device-specific sleep–wake scoring algorithms. Consequently, it is difficult to compare studies using different devices. While Meltzer et al. (2) have recently published recommendations for methodological details to include in protocols, it remains a challenge to establish standard practice parameters, even in adults (11).

There are a number of key sleep and wake actigraph variables, including sleep duration (24-h, nocturnal, and diurnal), daytime naps, and night-time wakings (number, duration, and distribution), the longest sleep period overnight (signaling sleep continuity), sleep efficiency, sleep latency, and sleep onset and offset times. Yet, to date, normative values for these variables remain patchy across the pediatric age range. Although many PSG validation studies have been conducted for overnight sleep across the pediatric age range (6), citing good sensitivity (sleep agreement) but poorer specificity (wake agreement), few consider 24-h validation. This is perhaps not surprising given the requirement for wired and connected EEG, but advances in EEG metrics within wireless sensing headsets may advance this area. Video data, at least could be used for 24-h validation with headgear and fixed video camera systems.

A major challenge is the requirement for parents to complete daily diaries or logs of accurate sleep onset and offset times, which must be manually entered into current scoring programs. Parental adherence to completion of 24-h sleep–wake diaries is problematic and made more difficult by having to adhere to this over several consecutive days. Acebo et al. (12) recommend seven consecutive days of recording to obtain five nights of useable data. However, Meltzer and Westin (13) provide evidence that actigraphy data collected without a diary still produces reliable data. Thus, in a comprehensive investigation of pediatric actigraphy scoring rules in 40 children using data collected over 7 days, they found no clinically meaningful differences related to several key sleep measures in the diary vs. no-diary condition. However, the no-diary condition precludes non-wear time from being identified accurately and some software require “time flags” (e.g., time in bed) for starting the algorithm.

Within infant and early childhood sleep, identification of daytime naps by actigraphy remains one of the most understudied areas of measurement. In their systematic review, Meltzer et al. (2) state that no guidelines exist for daytime naps and thus non-validated night-time rules have been used. Some programs with automatic scoring accept a minimum of 30 min of sleep to calculate sleep–wake summary parameters, making it difficult to accurately include short naps into a 24-h sleep pattern. Non-wear time during the day can also be misinterpreted as naps, as can events such as riding in the car with little movement. However, naps are not a problem specific to pediatric actigraphy. Berger et al. (11) highlight the lack of clear rules about evening naps in adults, prior to bedtime.

By convention, actigraphs are commonly placed on the non-dominant wrist for older children and on the ankle or calf for infants (14). While assessing sleep with physical activity, devices in children are typically worn around the waist for activity measures (15). However, many studies fail to report placement (2). It is unclear at what age the device should be placed on the wrist as opposed to the leg, or whether motor milestones, rather than age should be the cue. Simultaneous dominant and non-dominant wrist placement improves identification of artifacts mostly likely to be breathing-related (16), and the few studies that have examined waist vs. non-dominant wrist placement have reported no (17) or minimal (18) differences in total sleep time, or no differences in other main derived sleep parameters (19).

## Technical Basis of Actigraphy and Common Scoring Algorithms

Sleep–wake actigraphy devices operate on the basis that motion infers wakefulness, and that inactivity infers sleep. As such, the conventional actigraphy unit comprises an accelerometer-based motion sensor, a microprocessor and memory for data storage. During operation, such devices apply simple algorithms (time-above-threshold, zero-crossing, and digital integration) to summarize the overall intensity of measured accelerometry data across defined epochs (typically 15, 30, or 60 s) as “activity counts,” which are recorded on the device (3). The activity count data are processed offline by scoring from which wakefulness (high activity count) and sleep (low activity count) is inferred. Indeed, numerous algorithms have been developed to automatically score sleep and wake from raw activity data.

While various commercially available algorithms accompanying the different actimeters are available, the most commonly used algorithms in pediatric actigraphy, as reviewed by Meltzer et al. (2), are the Sadeh algorithm (16) and the algorithm of Cole et al. (20). Both algorithms were developed using wrist-worn ambulatory monitoring incorporation devices and validated in adults, and the Sadeh algorithm also validated in children and adolescents. Both were later adapted for use in other commercial devices. Typically, sleep algorithms vary by the population, device, and the site placement they were developed for (i.e., wrist, ankle, and waist), but most work in a similar fashion to define each minute of recorded activity (using a sliding window) as either a sleep or wake epoch by weighting the activity scores of the surrounding minutes. Problems arise where non-device-specific algorithms or placements are used, as different devices have different sensitivities, and placement influences output.

The Sadeh algorithm is computed as follows: PS = 7.601 − 0.065MW5 − 1.08NAT − 0.056SD6 − 0.073 ln ACT; where PS is the probability of sleep, MW5 is the average number of activity counts during the scored epoch and a window of five epochs preceding and following it, NAT is the number of epochs with an activity level of ≥50 but <100 activity counts per minute in an 11-min window, including the scored epoch and the five epochs preceding and following the scored epoch, SD6 is the standard deviation of the activity counts during the scored epoch and the five epochs preceding it, ln ACT is the natural logarithm of the number of activity counts during the scored epoch + 1; if PS is 0 or greater, the specific epoch is scored as sleep, otherwise it is scored as wake.

The Cole algorithm works in a similar fashion but instead of a sliding window of 5 min, it computes a weighted sum of the activity in the current minute, the preceding 4 min, and the following 2 min as follows: S = 0.0033(1.06an4 + 0.54an3 + 0.58an2 + 0.76an1 + 2.3a0 + 0.74a1 + 0.67a2); where an4–an1 are activity counts from the prior 4 min, a0 is the current minute, and a1 and a2 are the following 2 min. The current minute is scored as sleep when S < 1.

## Emerging Technologies to Improve Performance

Much of the fundamental technology underpinning actigraphy is now two decades old. As such, recent technological advances present new opportunities to improve the performance of actigraphy. In evaluating such advances, it is worth considering the nature of the errors in sleep/wake actigraphy scoring of sleep. The first are periods of sleep that include significant movements, erroneously classified as wake; the second are periods of wakefulness in which no movements are detected, erroneously classified as sleep. The latter can be further sub-divided into periods of genuine inactivity, and periods where movements are present, but not detected. The source of error in each case is different, and therefore, warrants different strategic approaches.

Using conventional actigraphy, epochs associated with sleep-related movements are likely to have high activity counts, and would therefore be misclassified as wake. However, if the nature of movements during sleep is characteristically different to the movements during wake (21), it may be possible to classify sleep and wake on the basis of the *type* of movement, rather than the intensity of activity (22–24). Unfortunately, there is limited opportunity to investigate such an approach using typical commercial systems due to the “on-device” algorithms that calculate epoch-by-epoch activity counts (25). Intrinsically, these algorithms summarize the information available in the raw accelerometry data, and consequently, characteristically different movements may be represented by identical activity counts.

Historically, this simple summarization step was essential due to the limited data storage available in a convenient watch-sized package. However, with modern microprocessors and flash memory, it is now technically possible to record unprocessed high temporal resolution accelerometry for extended periods. Indeed, such devices are now commercially available (25). Such technology could facilitate the application of more advanced algorithms to raw accelerometry data [such as those already applied in human activity monitoring (26–28)], to determine whether wake and sleep-related movements are characteristically different, and therefore, whether these differences may be exploited to improve the classification of epochs associated with sleep-related movements.

Until recently, the majority of commercially available actigraphy devices have used single-axis piezoelectric sensor elements to measure acceleration. Consequently, movements predominantly orthogonal to the sensing axis are difficult to detect, resulting in periods of apparently activity-free wake. However, with recent advances in manufacturing technology, current-generation devices have adopted micro-electro-mechanical systems (MEMS) based sensors (25), in which tri-axial accelerometers are able to be packaged in smaller sensors, and at lower cost. While few of these devices have been validated for pediatric sleep, a validation study comparing the tri-axial GTX3+ device to the uniaxial AW-64 device in healthy adults showed a higher epoch-by-epoch agreement in the tri-axial device, while the uniaxial device better estimated wake after sleep onset and total sleep time (29). It is unclear as to whether analysis tools have been developed to exploit the properties of tri-axial, and such developments remain an opportunity to improve actigraphy performance.

One explanation for actual movements that are not recorded is simply that the movement does not include the body part on which the actigraphy unit is located. An intuitive solution is the use of multiple actigraphy units strategically located on the body [i.e., upper limb, lower limb, and central body (22)]. To date, no published studies have investigated whether combining movements from multiple locations is able to improve actigraphy performance, possibly due to a range of practical and technical issues. First, one of the most appealing aspects of actigraphy is the simplicity of setup. The requirement for multiple units complicates this. Second, the ability to record synchronized activity at multiple locations is not typically available in commercial systems. Nonetheless, multi-site systems are routinely used in studies of human activity (30–32) indicating that this is not a technically insurmountable problem. Despite these potential challenges, multi-site accelerometry certainly warrants further investigation. In the clinical context, due consideration to the trade-off between performance and setup complexity is essential.

Periods of wakefulness corresponding to genuine inactivity are common in certain disorders of interest (i.e., insomnia), and may be particularly problematic in 24-h recordings, where significant periods of wakeful rest (i.e., watching television) may be present. At present, such periods are partially managed through the use of the Sadeh and Cole algorithms described above, which have the effect of “smoothing” epochs of activity to surrounding epochs – thus, an epoch of inactivity surrounded by periods of activity would be presumed to be a period of inactive wake (33). However, this has limitations in participants with highly fragmented sleep, and therefore, frequent short periods of wake and sleep.

Recent attempts have approached this problem by developing scoring models optimized for 24 h data (15), and with more complex stochastic modeling (34) and Markov model type approaches (35). While such approaches are more generalized, like the Sadeh and Cole algorithms, they place assumptions on the probability of certain sleep–wake progressions. Such assumptions may not be valid in physiological or pathological subgroups, or the individual subject. Nonetheless, investigation of algorithms customized for infants and young children – or strategies even for customizing algorithms in the individual – may yield significant performance benefits.

A technical approach that may address all error types is combining actigraphy with other relatively non-invasive physiological signals. Cardio-respiratory variables such as the respiratory pattern (from respiratory inductive plethysmography) and heart rate (from pulse oximetry or ECG) present good candidates due to their relative ease of application, and the fact they do not require cables around the face and neck (minimizing the risk of choking in children). Studies combining actigraphy with cardio-respiratory variables have been conducted in adults and children with promising results (24, 36–38). There is significant literature in adults, children, and infants (39–41), which demonstrate that cardio-respiratory variables can be used to discriminate sleep from wake, and indeed, provide the opportunity to sub-classify sleep as rapid eye movement (REM) and non-REM. While such combinations have the potential to improve performance and utility, there are significant trade-offs in terms of study complexity and invasiveness. Such an approach may be most applicable to minimal channel studies to investigate sleep disorders where cardio-respiratory variables are already measured.

Smartphone apps for actigraphy have become widely available over the last few years with the accelerometer built into the mobile phone or an external device. However, a 2013 review of devices on the market found no apps provided scientific backup information or algorithm details (42). Validations have been attempted against Actiwatch in two very small studies using different devices, one reporting reasonable agreement with total sleep time, wake after sleep onset and sleep efficiency, but not sleep onset latency (43), the other reporting good agreement regarding sleep efficiency (44). As far as we are aware, no smartphone devices have been validated against PSG.

## Combining Sleep and Physical Activity

Some researchers in the physical activity field are beginning to report on sleep. Physical activity devices (core accelerometers) have optimal hip placement (45) and use sensors orientated and more sensitive to vertical acceleration associated with walking/running (46), whereas for sleep the sensor orientation is more horizontal intended to be more sensitive to hand movements. Output data from core accelerometers include activity intensity categorized into various levels of sedentary, light, and moderate physical activity making them extremely valuable tools for assessing relative levels of physical activity in health and disease.

We and others have expanded the use of this technology to integrate sleep and physical activity measures within the one device (15, 47, 48). Our work has focused on developing a new count-scaled algorithm and MATLAB™ script that produces outputs demonstrated to enhance the utility and accuracy of both sleep (48) and activity measures (49). The scaling process standardizes counts across the entire recording, which gives the algorithm flexibility to apply to other accelerometers where count outputs differ from different sensor sensitivities, or placements. The algorithm is integrated into a MATLAB™ script programed to detect sleep onset (night-time sleep) and sleep offset (morning wake) and all sleep and wake epochs in between as computed and compared to a sleep–wake threshold. Standard sleep and wake variables (e.g., sleep minutes and number of wakings) are calculated for all periods between falling asleep and waking. Key physical activity variables (sedentary and moderate to vigorous activity) are then scored for all periods between waking and falling asleep. The major advantage is that sleep onset and offset for different participants is individualized. In addition, automated batch-scoring can be applied providing a major time advantage over other commercially available software programs for which sleep filters for each day and for each participant have to be applied prior to scoring. We have recently shown that failure to remove sleep prior to physical activity scoring can markedly affect estimates of counts per minute, wear time, and sedentary time (49), whereas our MATLAB™ script and count-scaled algorithm produces comparable estimates of both sleep and activity (49) to other, more labor intensive methods (15). However, further research is required to demonstrate the value of the algorithm and of integrating both sleep and physical activity measurements in large datasets and over different developmental ages.

## Conclusion

Currently, the actigraphy field lacks established standards for pediatric sleep measurement (2). While this also applies to the adult field (11), pediatric sleep presents unique challenges, particularly in the very young with often fragmented overnight sleep, and several sleep periods over 24-h. Scoring rules have never been considered for naps, and the reliance on parental diaries to report non-wear time and sleep onset and offset times is problematic. Furthermore, no normative values for key sleep variables are available across the pediatric age range, severely hampering access to reference standards for clinicians and researchers alike. In 2006, Acebo and LeBourgeois (50) recommended scoring rules for sleep should be established *a priori*, but no advances have been made. We reiterate the call made by Meltzer and Westin (13) that, until established standards for the scoring and reporting of actigraphy data are in place, researchers need to be conscientious when using actigraphy and clearly report scoring rules and variables.

Finally, many of the problems facing accuracy of measurement are inherent within the field of actigraphy itself, particularly within sleep–wake classification. Actigraphy cannot possibly replace the preciseness of information the neurophysiological signals of PSG reveal about the timing of sleep onset and offset, and in particular sleep fragmentation. This raises the question as to whether or not PSG sets too high a standard against which to validate actigraphy studies. We suggest researchers engaged in algorithm development could consider discriminating the type of movement occurring during sleep, as being distinct from wake, through more sophisticated processing techniques. Results from emerging research, such as multi-site application to measure movements from different parts of the body, or actigraphy combined with other non-invasive physiological cardio-respiratory signals, may pave the way for advances in algorithm development to enhance sensitivity and accuracy. Devices that record raw tri-axial accelerometry data for extended periods are now available, presenting an opportunity to directly compare conventional and novel processing and scoring algorithms in identical data. This has the potential to overcome problems associated with comparing studies using different proprietary devices.

## Conflict of Interest Statement

The authors declare that the research was conducted in the absence of any commercial or financial relationships that could be construed as a potential conflict of interest.
